# Which score best predicts new-onset AF after primary PCI in STEMI? Head-to-head assessment of four algorithms

**DOI:** 10.3389/fcvm.2026.1835662

**Published:** 2026-10-21

**Authors:** Xiaofeng Zhou, Hailiang Ma, Zhenhua Jiang

**Affiliations:** Department of Cardiology, Shaoxing Central Hospital, Shaoxing, Zhejiang, China

**Keywords:** atrial fibrillation, mC2HEST score, primary PCI, risk prediction, STEMI

## Abstract

**Background:**

New-onset atrial fibrillation (NOAF) is a frequent and clinically significant comorbidity following primary percutaneous coronary intervention (PCI) for ST-segment elevation myocardial infarction (STEMI), associated with increased mortality, stroke risk, and healthcare costs. Predicting NOAF in this setting remains challenging. This study aimed to compare the predictive performance of four risk scores—CHA_2_DS_2_-VASc-60, mC2HEST, Mayo-AF, and HATCH—in identifying patients at high risk of NOAF after primary PCI in STEMI.

**Methods:**

We conducted a single-center, retrospective cohort study at Shaoxing Central Hospital, including consecutive adults with STEMI who underwent primary PCI within 12 h of symptom onset between February 2020 and June 2025. Patients with a history of atrial fibrillation or other exclusions were excluded. The endpoint was NOAF within 1 year after PCI, documented by continuous inpatient telemetry, 12-lead ECG at discharge, and routine follow-up ECGs at 1, 3, 6, 9 and 12 months. We compared the discriminative ability of the four scores using receiver operating characteristic (ROC) curves and assessed their clinical utility through net reclassification improvement (NRI) and integrated discrimination improvement (IDI).

**Results:**

A total of 1,175 patients were included in the final analysis. The incidence of NOAF within 1 year was 8.2% (*n* = 96). The mC2HEST score demonstrated the highest area under the ROC curve (AUC) for predicting NOAF (AUC = 0.786), followed by HATCH (AUC = 0.746) and CHA_2_DS_2_-VASc-60 (AUC = 0.739), while Mayo-AF had the lowest AUC (AUC = 0.736).

Pairwise comparisons showed that mC2HEST significantly outperformed CHA_2_DS_2_-VASc-60 (*Δ*AUC = 0.046, *p* = 0.002).

**Conclusions:**

Among the four risk scores evaluated, mC2HEST provided the best predictive performance for NOAF after primary PCI in STEMI patients. Its superior discriminative ability and significant integrated discrimination improvement suggest that mC2HEST could be a valuable tool for identifying high-risk patients who may benefit from intensified monitoring and early interventions. Future prospective, multicenter studies are warranted to validate these findings.

## 1 Introduction

Primary percutaneous coronary intervention has transformed ST-segment elevation myocardial infarction into a largely survivable event; yet, within the first 48 h of reperfusion, approximately one in ten patients develops new-onset atrial fibrillation (NOAF), characterized by rapid and irregular atrial activity. This early arrhythmia is not a benign epiphenomenon: it reflects abrupt left atrial stretches, calcium overload, oxidative burst and sympathetic surge that convert ischemic myocardium into a proarrhythmic substrate. Importantly, new-onset atrial fibrillation peaks precisely when platelet activation, endothelial dysfunction and hypercoagulability are maximal, amplifying the likelihood of systemic embolism and microvascular compromise (1–3).

Population-based registries and individual patient-level metanalyses consistently demonstrate that even transient new-onset atrial fibrillation doubles in-hospital mortality and triples 30-day ischemic stroke risk. Longterm follow-up reveals a 1.5-fold higher 5-year all-cause mortality and a twofold increase in repeat heart failure hospitalizations. Length of stay expands by a median of 2.5 intensive care days and four total hospital days, pushing incremental per patient costs above $7,500 and consuming an estimated 1% of total acute MI budgets in high-income economies (3–5). Crucially, new-onset atrial fibrillation clusters in elderly, anterior-wall or microvascular dysfunction phenotypes, thereby widening outcome inequalities.

Despite its clinical footprint, new-onset atrial fibrillation prediction in ST-segment elevation myocardial infarction remains rudimentary. Community derived algorithms underestimate risk in younger patients with large infarct size while overestimating risk in older patients with minor myocardial injury. Within the acute coronary syndrome spectrum, prior studies have evaluated individual scores or compared them in mixed ACS/PCI cohorts (6, 7), and HATCH has been validated in STEMI populations (8–11). However, a head-to-head comparison of CHA_2_DS_2_-VASc-60, mC2HEST, Mayo-AF, and HATCH in a pure STEMI primary-PCI cohort with 1-year follow-up remains lacking (12). Consequently, clinicians default to clinical judgment or generic scores, resulting in inconsistent monitoring and missed opportunities for early intervention.

Each candidate algorithm interrogates a distinct pathophysiological axis: CHA_2_DS_2_-VASc-60 emphasizes thromboembolic substrate; mC2HEST captures pulmonary, metabolic and thyroid drivers; Mayo-AF weights valvular atrial remodeling; and HATCH focuses on systemic vascular and cerebrovascular burden (13–17). Direct, simultaneous comparison within a single ST-segment elevation myocardial infarction cohort will clarify which tool, or parsimonious combination, optimally stratifies patients for intensified rhythm monitoring, early antiarrhythmic strategies or prolonged anticoagulation—while avoiding unnecessary resource utilization in low-risk individuals.

We therefore conducted a large scale, head-to-head evaluation of the discriminative performance, reclassification capacity and clinical net benefit of CHA_2_DS_2_-VASc-60, mC2HEST, Mayo-AF and HATCH scores for predicting 1-year new-onset atrial fibrillation after primary percutaneous coronary intervention in ST-segment elevation myocardial infarction.

## 2 Methods

### 2.1 Study design and setting

We conducted a single center, retrospective cohort study at Shaoxing Central Hospital, a 2,000 beds tertiary academic PCI referral center and certified level III Chest Pain Unit in Zhejiang Province, eastern China. The hospital serves approximately 4 million residents and performs approximately 2,000 primary PCIs annually. Consecutive patients who underwent emergent percutaneous coronary intervention for ST segment elevation myocardial infarction between February 2020 and February 2025 were identified from the Cath Lab database. This registry captures real-time procedural, angiographic and outcome data for all coronary interventions performed within the institution. The study was designed as an observational cohort analysis; Reporting follows the STROBE statement for retrospective analyses.

### 2.2 Ethics and reporting

The study protocol was reviewed and approved by the Institutional Review Board of Shaoxing Central Hospital (Ref. 2025IRB062). All procedures were conducted in accordance with the Declaration of Helsinki and local regulatory requirements. Need for informed consent was waived by the Institutional Review Board of Shaoxing Central Hospital. Diagnostic codes were extracted from the hospital's electronic health record system using the International Classification of Diseases, 10th Revision (ICD10) and 9th Revision, Clinical Modification (ICD9CM) classifications. Reporting follows the Strengthening the Reporting of Observational Studies in Epidemiology (STROBE) statement for retrospective cohort analyses.

### 2.3 Participants

#### 2.3.1 Inclusion criteria

We enrolled consecutive adults (≥18 years) with a first ever diagnosis of ST Segment elevation myocardial infarction according to the Fourth Universal Definition of Myocardial Infarction (18), who underwent primary percutaneous coronary intervention within 12 h of first medical contact and were admitted directly through emergency medical services or the institutional emergency department (2).

#### 2.3.2 Exclusion criteria

Patients were excluded if they had any documented history of atrial fibrillation (including paroxysmal AF) or atrial flutter, prior atrial fibrillation ablation, long-term antiarrhythmic therapy for atrial fibrillation, valvular atrial fibrillation, moderate to severe native cardiac valve disease, active hyperthyroidism (on antithyroid medication or TSH < 0.1 mIU/L within 6 months), or uninterpretable baseline electrocardiograms.

### 2.4 Data sources and variables

Data were extracted from the hospital's electronic health record and the institutional Cath-Lab database. Clinical characteristics used for score calculation were assessed at admission, before primary PCI, and included age, sex, body mass index, cardiovascular risk factors, medications, and first-medical-contact-to-device time. Post-procedural laboratory results (drawn at 6AM on the morning following PCI) included hemoglobin, creatinine, lipid profile, and B-type natriuretic peptide; these were used for descriptive comparison only and were not included in risk score calculations. Quantitative coronary angiography data were also documented.

The overall missing rate for key variables and all score components was 2% (uniformly below 3% across individual components), and a complete-case analysis was performed with patients with missing data excluded.

### 2.5 Score calculation

Four established risk scores—CHA_2_DS_2_-VASc-60, mC2HEST, Mayo-AF, and HATCH—were selected because they are all bedside-calculable using routinely available clinical variables, have been validated for incident atrial fibrillation (AF) prediction in cardiovascular or general populations, and collectively cover distinct pathophysiological axes relevant to post-infarction arrhythmogenesis (thromboembolic substrate, metabolic/thyroid drivers, valvular remodeling, and systemic vascular/cerebrovascular burden). None was originally derived for STEMI primary-PCI cohorts: CHA_2_DS_2_-VASc-60 was modified from the stroke-risk CHA_2_DS_2_-VASc score (16), mC2HEST from a primary-care incident-AF cohort (15), Mayo-AF from a general clinical population (11), and HATCH from AF-progression risk stratification (17). This gap is precisely why a head-to-head external validation in this high-risk population is clinically warranted.

CHA_2_DS_2_-VASc-60 (19).

congestive heart failure or left ventricular ejection fraction (LVEF) ≤ 40% (1 point), hypertension (1 point), diabetes (1 point), vascular disease (1 point), female sex (1 point), age 60–64 years (1 point), stroke/transient ischemic attack (TIA)/thromboembolism (2 points), and age ≥65 years (2 points). The total score ranges from 0 to 9 points.

mC2HEST (15).

coronary artery disease (1 point), chronic obstructive pulmonary disease (COPD) (1 point), hypertension (1 point), hyperthyroidism (1 point), systolic heart failure (2 points), age 65–74 years (1 point), and age ≥75 years (2 points). The total score ranges from 0 to 8 points.

Mayo-AF (11).

heart failure (3 point), valvular disease (2 point), coronary artery disease (CAD) (2 point), hypertension (1 point), diabetes (1 point), male sex (1 point), age below 65 years (0 point), age 65–75 years (1 points), and age above 75 years (2 points). The total score ranges from 0 to 12 points. In this cohort, valvular disease was uniformly absent (0 points) due to pre-specified exclusion criteria.

HATCH (20).

hypertension (1 point), age ≥75 years (1 point), COPD (1 point), heart failure (2 points), and stroke/TIA (2 points). The total score ranges from 0 to 7 points.

All components were operationalized according to each score's original derivation criteria; heart failure definitions varied slightly across scores (CHA_2_DS_2_-VASc-60: prior HF or LVEF ≤ 40%; mC2HEST: systolic HF; Mayo-AF/HATCH: clinical HF).

### 2.6 Follow-up and endpoint assessment

This was a retrospective cohort study. Follow-up ECG assessments were conducted according to a prespecified protocol: continuous inpatient telemetry until discharge, a 12-lead ECG at discharge, and routine outpatient 12-lead ECGs at 1, 3, 6, 9, and 12 months post-PCI. All outpatient ECGs were performed during scheduled hospital clinic visits. NOAF was defined as any episode of atrial fibrillation documented by telemetry or 12-lead ECG during the 1-year follow-up period.

The primary endpoint was new-onset atrial fibrillation (NOAF) within 1 year after primary PCI. NOAF was diagnosed according to standard ECG criteria (irregularly irregular rhythm without discernible P waves) with a minimum duration of ≥30 s. Atrial flutter was included as part of the NOAF endpoint. Episodes detected only by inpatient telemetry were also included if they met the above duration and ECG criteria. Thirty-day AF incidence was analyzed as a secondary exploratory outcome.

### 2.7 Sample size justification

We assumed a conservatively observed NOAF rate of 8% based on our 2022 institutional audit (21, 22). To detect a clinically relevant 0.05 difference in AUC between two correlated risk scores with 80% power at *α* = 0.05 (two-sided), the method of DeLong requires ≥72 events.1 An event yield of 8% therefore mandates a minimum of 900 eligible patients. Accounting for 5% missing covariates, we targeted 1,100 patients, anticipating ≈88 events.

### 2.8 Statistical analysis

All analyses were performed with SPSS Statistics 27.0 (IBM Corp., Armonk, NY). Normally distributed data are presented as mean ± SD and were compared with the independent sample test; non-normally distributed data are expressed as median (Q1, Q3) and were compared with the Mann–Whitney *U*-test. Categorical variables are reported as *n* (%) and were compared using the *χ*^2^-test or Fisher's exact test as appropriate.

ROC curves were constructed to assess the ability of each score to predict NOAF. AUCs and their 95% confidence intervals (CIs) were calculated using the DeLong method. Paired AUC differences from the same patients were compared with the DeLong test, with 95% CIs derived from the DeLong standard error. NRI and IDI were calculated using category-based reclassification with tertiles of the reference model's predicted 1-year NOAF probabilities, derived from univariable logistic regression models fitted to each score; the baseline event rate was incorporated through the logistic model intercept. Their 95% CIs were obtained via 2,000 bootstrap resamples. Given the sensitivity of these measures to model specification, results were interpreted alongside AUC and calibration metrics. CHA_2_DS_2_-VASc-60 was prespecified as the reference model for all AUC, NRI, and IDI comparisons. Given this prespecified many-to-one design and the limited number of comparisons (three), no formal adjustment for multiple comparisons was performed; all exact *P*-values are reported for transparency.

## 3 Results

### 3.1 Flow chart

Of 2,847 consecutive patients in the Cath-Lab database, 1,650 were excluded (prior/valvular AF: 194; presentation >12 h: 1,445; uninterpretable ECG: 11), leaving 1,197 eligible patients. 22 were lost to ECG follow-up, yielding a final analytic cohort of 1,175 patients (96 NOAF; 1,079 no AF) (Figure 1).

**Figure 1:**
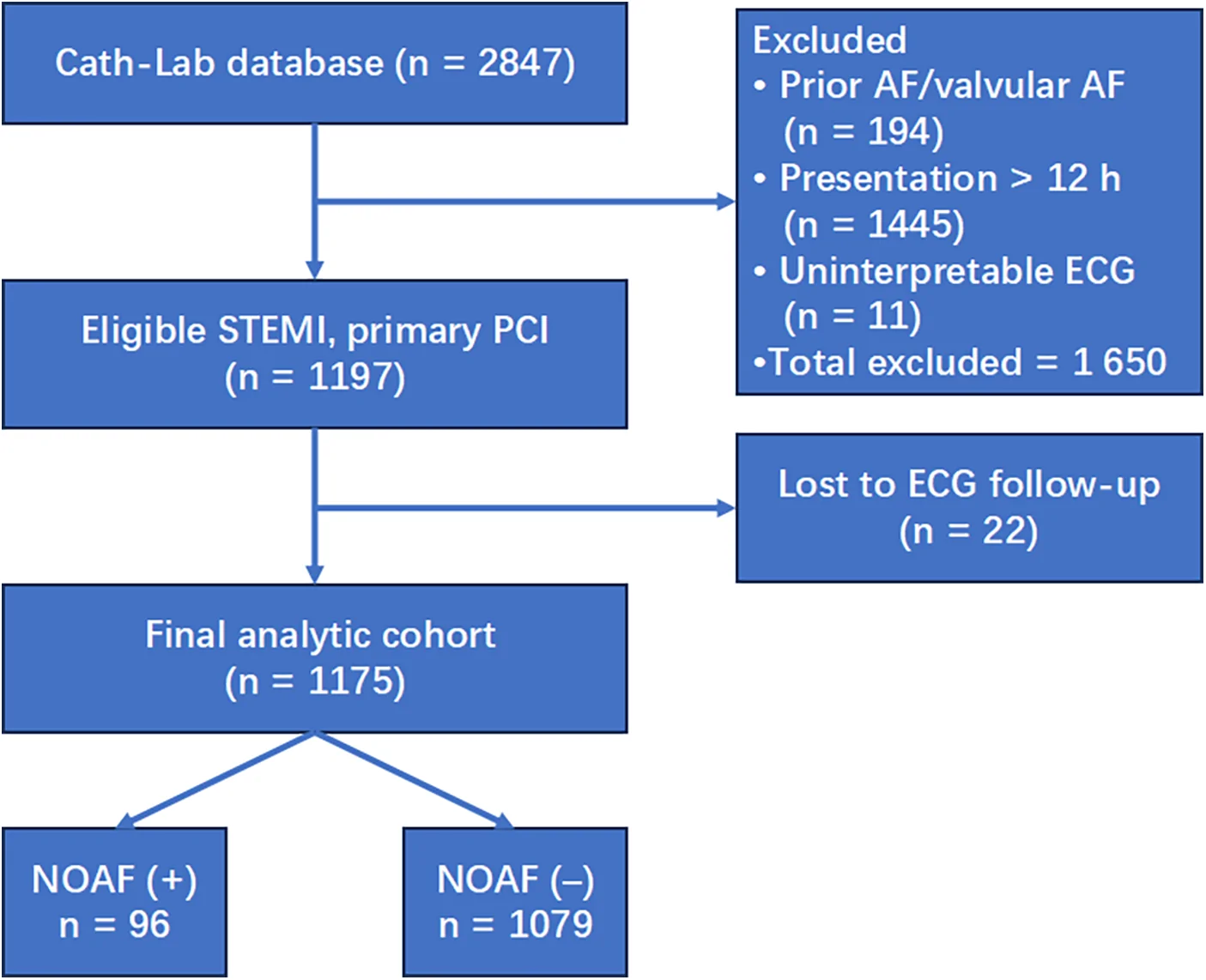
STROBE flow diagram of study participants.

### 3.2 Cohort characteristics

Table 1 summarizes the baseline features of the 1,175-patient analytic cohort (median age 63 years). Individuals who developed new-onset atrial fibrillation were significantly older (*p* < 0.001), more frequently hypertensive (*p* = 0.018) and had a higher prevalence of pre-existing heart failure (*p* < 0.001) than those without NOAF. Admission B-type natriuretic peptide concentration was also higher in the NOAF group (*p* < 0.001). Sex distribution, body-mass index, diabetes status, lipid profile, renal function did not differ between groups (all *p* > 0.05).

**Table 1:** Baseline characteristics by NOAF status.

Variable	Non-NOAF (*n* = 1 079)	NOAF (*n* = 96)	*P* value
Demographics			
Age, y	62.1 ± 6.1	70.4 ± 5.8	0.000
Female sex, *n* (%)	264 (24.5)	28 (29.2)	0.184
BMI, kgm⁻^2^	23.8 ± 3.0	23.5 ± 2.5	0.288
Current smoking	374 (34.7)	29 (30.2)	0.222
Comorbidities			
Hypertension, *n* (%)	618 (57.3)	66 (68.8)	0.018
Diabetes mellitus, *n* (%)	322 (29.8)	30 (31.3)	0.427
COPD, *n* (%)	97 (9.0)	12 (12.5)	0.169
Heart failure, *n* (%)	237 (22.0)	49 (51.0)	0.000
Stroke/TIA, *n* (%)	89 (8.2)	8 (8.3)	0.547
PVD, *n* (%)	35 (3.2)	4 (4.2)	0.397
History of hyperthyroidism, *n* (%)	7 (0.6)	2 (2.1)	0.163
Biochemical Indicators			
Hemoglobin, g L⁻^1^	134.7 ± 8.7	133.1 ± 9.7	0.089
TG (mmol/L)	0.93 (0.79,1.09)	0.90 (0.75,0.93)	0.523
TC (mmol/L)	4.51 ± 1.13	4.38 ± 1.37	0.320
HDL-C (mmol/L)	1.18 ± 0.29	1.21 ± 0.30	0.302
LDL-C (mmol/L)	3.33 ± 0.79	3.23 ± 0.43	0.194
Scr (*μ*mol/L)	81.2 ± 19.4	78.5 ± 25.8	0.195
BNP (ng/mL)	223 (174,276)	294 (236, 367)	0.000

BMI, body-mass index; COPD, chronic obstructive pulmonary disease; TIA, transient ischemic attack; PVD, peripheral vascular disease; TG, triglycerides; TC, total cholesterol; HDL-C, high-density lipoprotein cholesterol; LDL-C, low-density lipoprotein cholesterol; Scr, serum creatinine; BNP, B-type natriuretic peptide.

### 3.3 Angiographic findings, reperfusion times and medications

Infarct-related artery distribution differed between groups (*p* = 0.008): the left-anterior-descending artery was the culprit in 74.0% of patients with subsequent NOAF vs. 58.1% of those without (Table 2). Pre-procedural TIMI 0/1 flow was more common in the NOAF cohort (*p* = 0.003), whereas final TIMI-3 flow rates were similar (>93%). First-medical-contact-to-balloon time was comparable (median 75 min; IQR 68–83). Glycoprotein IIb/IIIa inhibitor use was higher in patients who developed NOAF (51.0% vs. 38.1%; *p* = 0.009), as was peri-procedural loop-diuretic therapy (*p* = 0.008). ACE-I/ARB and β-blocker prescription rates were similar between groups (all *p* > 0.05).

**Table 2:** Angiographic and pharmacologic profile by NOAF status.

Variable	Non-NOAF (*n* = 1 079)	NOAF (*n* = 96)	*P* value
IRA, *n* (%)			0.008
LAD	627 (58.1)	71 (74.0)	
LCX	158 (14.6)	11 (11.5)	
RCA	294 (25.5)	14 (14.6)	
Diseased vessels, *n* (%)			0.207
Single-vessel	618 (57.3)	46 (47.9)	
Two-vessel	320 (29.7)	35 (36.5)	
Three-vessel	141 (13.1)	15 (15.6)	
Pre-PCI TIMI flow, *n* (%)			0.003
0/1	775 (71.8)	84 (87.5)	
2	214 (19.8)	10 (10.4)	
3	90 (8.3)	2 (2.1)	
Post-PCI TIMI flow, *n* (%)			0.506
0/1	18 (1.7)	3 (3.1)	
2	47 (4.4)	3 (3.1)	
3	1 014 (94.0)	90 (93.8)	
FMC-B (min)	75 (68,83)	74 (61,87)	0.211
medications, *n* (%)			
GPI	411 (38.1)	49 (51.0)	0.009
ACE-I/ARB	923 (85.5)	80 (83.3)	0.323
*β*-blocker	746 (69.1)	59 (61.5)	0.077
Loop diuretic	159 (14.7)	24 (25.0)	0.008

IRA, Infarct-related artery; FMC-B, FMC-to-balloon time; GPI, Glycoprotein IIb/IIIa inhibitor; ACE-I/ARB, Angiotensin-Converting Enzyme Inhibitor/Angiotensin Receptor Blocker.

### 3.4 NOAF incidence across risk score groups

Figure 2 illustrates the incidence of new-onset atrial fibrillation (NOAF) across the full range of risk scores for the CHA_2_DS_2_-VASc-60, mC2HEST, Mayo-AF, and HATCH scoring systems.

**Figure 2:**
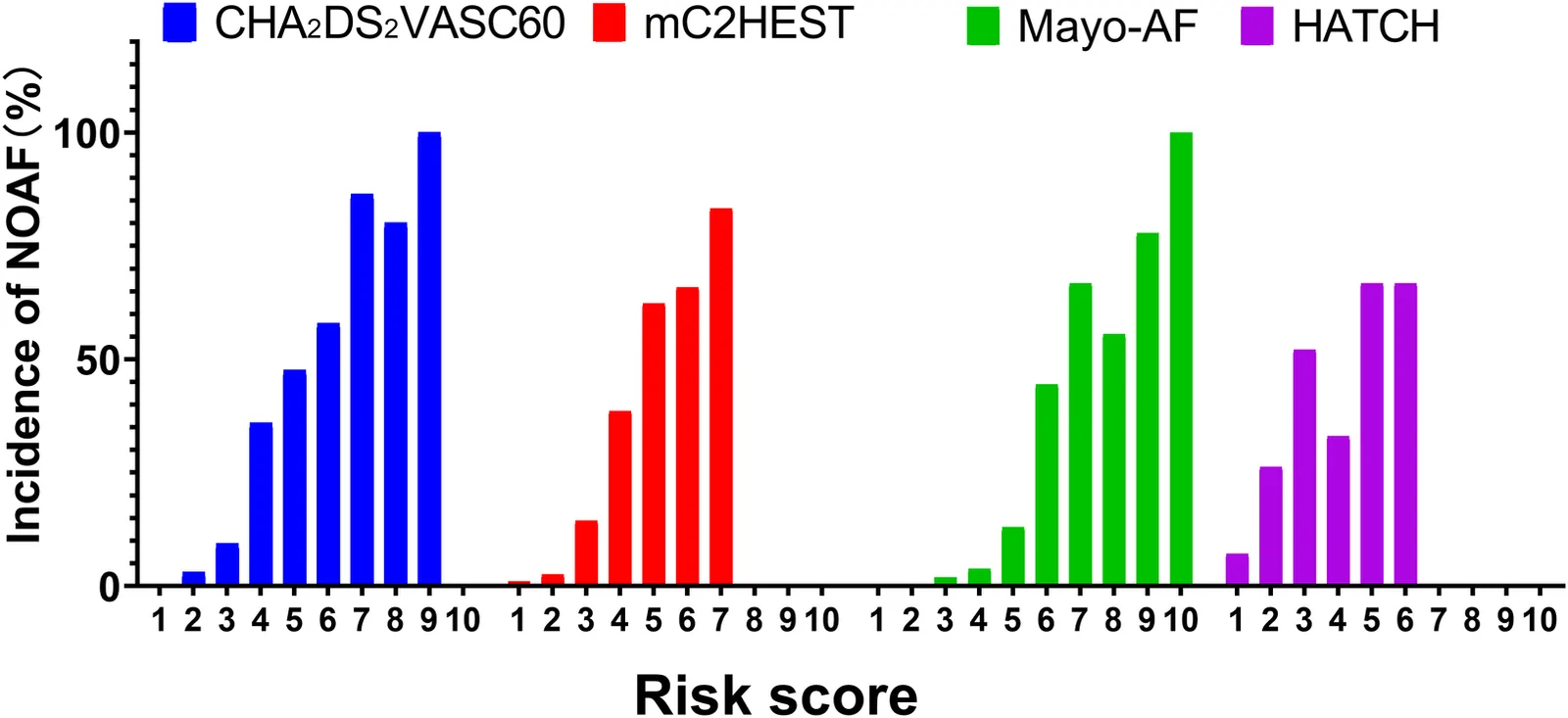
NOAF incidence across different risk score categories.

For the CHA_2_DS_2_-VASc-60 score, NOAF incidence rose progressively from 0.4% at 1 point to 100% at 9 points. In the mC2HEST score, incidence increased from 1.1% at 1 point to 83.3% at 7 points. The Mayo-AF score remained at 0% through 2 points, then climbed steadily to 66.7% at 7 points, with a slight dip at 8 points (55.5%) before reaching 100% at 10 points. For the HATCH score, incidence increased from 7.1% at 1 point to a plateau of 66.7% at 5–6 points.

### 3.5 Discrimination

Among the four scores evaluated, mC2HEST achieved the highest area under the receiver-operating characteristic curve (AUC) for predicting 1-year NOAF, with a value of 0.786 (Table 3 and Figure 3). HATCH and CHA_2_DS_2_-VASc-60 showed intermediate performance (AUC 0.746 and 0.739, respectively), whereas Mayo-AF yielded the lowest AUC (0.736).

**Table 3:** ROC-AUC estimates.

Risk score	AUC (95% CI)	Sensitivity at optimal cut-off	Specificity at optimal cut-off	Youden's Index
Mayo-AF	0.736 (0.691–0.780)	0.792	0.663	0.454
HATCH	0.746 (0.706–0.786)	0.656	0.780	0.437
mC2HEST	0.786 (0.747–0.824)	0.875	0.651	0.526
CHA_2_DS_2_-VASc-60	0.739 (0.704–0.775)	0.917	0.532	0.449

**Figure 3:**
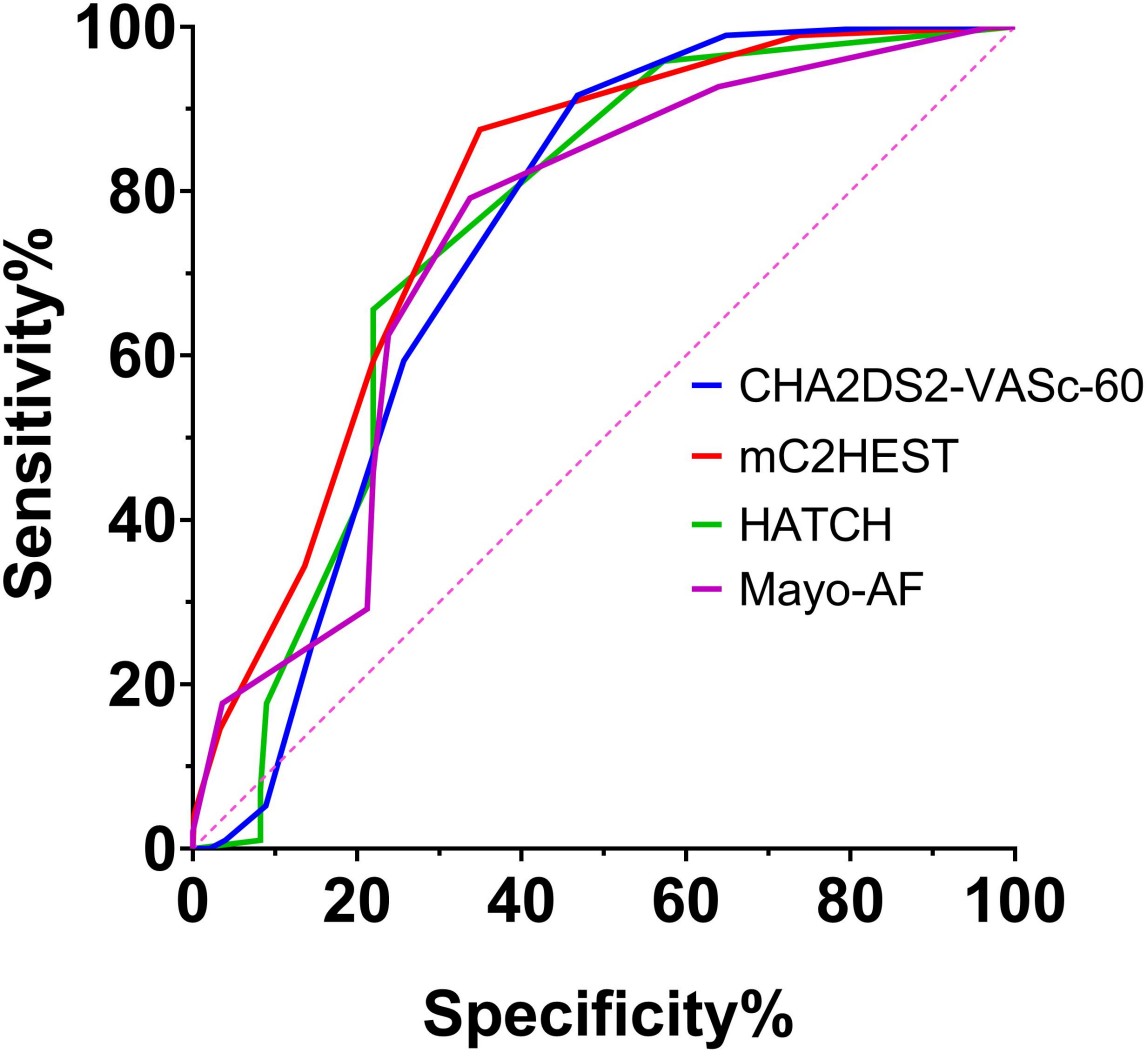
ROC curves for predicting new-onset atrial fibrillation using CHA_2_DS_2_-VASc-60, mC2HEST, HATCH scores and mayo-AF.

### 3.6 Delong tests

Pairwise comparisons against the CHA_2_DS_2_-VASc-60 reference are displayed in Table 4. mC2HEST exhibited a statistically higher AUC (Δ = 0.046,*p* = 0.002). In contrast, neither Mayo-AF (Δ = −0.003, *p* = 0.873) nor HATCH (Δ = 0.007, *p* = 0.654) achieved a meaningful increment in discrimination over CHA_2_DS_2_-VASc-60.

**Table 4:** Pairwise deLong tests: discrimination of alternative scores vs. CHA_2_DS_2_-VASc-60 for NOAF.

Comparison	*Δ*AUC (95% CI)	Z	*P* value
mC2HEST vs. CHA_2_DS_2_-VASc-60	0.046 (0.017–0.076)	3.096	0.002
Mayo-AF vs. CHA_2_DS_2_-VASc-60	−0.003 (−0.044 to 0.034)	−0.160	0.873
HATCH vs. CHA_2_DS_2_-VASc-60	0.007(−0.023 to 0.037)	0.449	0.654

### 3.7 Reclassification and integrated discrimination gains relative to CHA_2_DS_2_-VASc-60

Compared with the reference CHA_2_DS_2_-VASc-60 score (Table 5), mC2HEST demonstrated a statistically significant improvement in IDI (0.056, 95% CI 0.029–0.090; *P* < 0.001). Mayo-AF also achieved a significant IDI (0.022, 95% CI 0.006–0.038; *P* = 0.007), whereas HATCH did not (IDI = −0.003, 95% CI −0.013 to 0.007; *P* = 0.469). In contrast, category-based NRI did not reach statistical significance for any of the three scores (mC2HEST: 0.075, *P* = 0.419; Mayo-AF: 0.019, *P* = 0.813; HATCH: −0.028, *P* = 0.766).

**Table 5:** NRI and IDI relative to CHA_2_DS_2_-VASc-60.

Comparison model	Overall NRI (95% CI)	*p*-value	NRI in events	NRI in non-events	IDI (95% CI)	*p*-value
mC2HEST	0.075(−0.025 to 0.325)	0.419	−0.354	0.429	0.056 (0.029–0.090)	<0.001
Mayo-AF	0.019 (−0.137 to 0.175)	0.813	−0.031	0.050	0.022 (0.006–0.038)	0.007
HATCH	−0.028(−0.288 to 0.073)	0.766	−0.271	0.243	−0.003(−0.013 to 0.007)	0.469
CHA_2_DS_2_-VASc-60	Reference	–	–	–	Reference	–

### 3.8 Secondary analysis: 30-day atrial fibrillation rates

Four scores were evaluated for 30-day AF risk stratification (Table 6). Mayo-AF and mC2HEST produced the steepest gradients, with the highest odds ratios (Mayo-AF: OR = 5.54, 95% CI 3.21–9.56; mC2HEST: OR = 5.39, 95% CI 3.21–9.06). CHA_2_DS_2_-VASc-60 achieved intermediate stratification (OR = 2.04, 95% CI 1.15–3.64). HATCH did not significantly discriminate 30-day AF risk (OR = 1.15, 95% CI 0.48–2.74; *P* = 0.646).

**Table 6:** Secondary outcome: risk-stratified 30-day atrial fibrillation incidence and odds ratios across four scores.

Score	Stratum	Atrial fibrillation/total (%)	OR (95% CI)	*P* value
CHA_2_DS_2_-VASc-60	>4 vs. ≤4	17/181 (9.4) vs. 48/994 (4.8)	2.04 (1.15–3.64)	0.020
mC2HEST	>3 vs. ≤3	40/294 (13.6) vs. 25/881 (2.8)	5.39 (3.21–9.06)	<0.001
Mayo-AF	>5 vs. ≤5	42/317 (13.2) vs. 23/858 (2.7)	5.54 (3.21–9.56)	<0.001
HATCH	>4 vs. ≤4	6/96 (6.2) vs. 59/1,079 (5.5)	1.15 (0.48–2.74)	0.646

## 4 Discussion

New-onset atrial fibrillation (NOAF) is increasingly recognized as a long-term, modifiable cardiovascular risk modifier rather than a transient epiphenomenon of acute myocardial infarction (21). By extending follow-up to 12 months and using protocol-mandated outpatient electrocardiograms, the present study provides the first dedicated comparison of four bed-side risk scores for predicting NOAF after primary percutaneous coronary intervention (PCI) for ST-segment elevation myocardial infarction (STEMI). We demonstrate that the mC2HEST algorithm achieves superior discrimination, improved integrated discrimination and sustained prognostic separation over 12-month follow-up period, outperforming the traditional CHA_2_DS_2_-VASc-60, comparisons with Mayo-AF and HATCH did not reach statistical significance. These findings may help inform risk stratification for monitoring intensity and highlight the need for further studies evaluating anti-arrhythmic and anticoagulation strategies in high-risk patients (23, 24).

The primary observation is that, compared with CHA_2_DS_2_-VASc-60, an incremental AUC of 0.046 for mC2HEST is accompanied by a statistically significant **integrated discrimination improvement (IDI) of 0.056** (95% CI 0.029–0.090; *P* < 0.001), confirming enhanced separation of event and non-event predicted probabilities. Of note, category-based net reclassification improvement (NRI) did not reach statistical significance (0.075, 95% CI −0.025–0.325; *P* = 0.419). This discrepancy likely reflects the inherent sensitivity of category-based NRI to the choice of risk thresholds: using tertiles of the reference model's predicted probabilities confers an inherent classification advantage to the reference score, which may attenuate the apparent NRI of competing models. The more robust IDI, which evaluates continuous predicted probabilities without arbitrary categorization, consistently demonstrates superior discrimination for mC2HEST (25–27). Mechanistically, mC2HEST incorporates coronary artery disease, systolic heart failure, chronic obstructive pulmonary disease and hyperthyroidism—factors that converge on left-atrial stretch, oxidative burst and adrenergic surge during the vulnerable reperfusion window. Age is weighted exponentially (≥75 years = 2 points), paralleling the exponential rise in cytosolic calcium transient heterogeneity and atrial fibrosis density seen in elderly myocardium. By simultaneously encoding thrombotic, metabolic and neuro-humoral axes, mC2HEST supplies electrophysiological information not captured by CHA_2_DS_2_-VASc-60, which is confined to thrombo-embolic substrates (28, 29).

The performance of mC2HEST across 12 months is noteworthy. Previous models were derived from heterogeneous acute coronary syndrome cohorts or stable elective PCI populations and rarely followed patients beyond index hospitalization (30–32). Our prospective maintenance of a dedicated STEMI registry with scheduled outpatient ECGs at 1, 3, 6, 9, and 12 months minimizes surveillance bias and demonstrates that the score's discriminative signal persists into the chronic remodeling phase. This longitudinal stability implies that the biological drivers captured by mC2HEST—atrial fibrosis, diastolic stiffness, autonomic imbalance—continue to influence arrhythmogenesis long after the acute infarct has healed. Therefore, the score may help identify patients who could benefit from more intensive in-hospital monitoring and closer outpatient follow-up; whether score-guided anticoagulation decisions improve outcomes requires prospective validation (33).

Prior studies have validated individual AF-risk scores in ACS or STEMI populations—HATCH in STEMI, mC2HEST in mixed ACS-PCI cohorts, and CHA_2_DS_2_-VASc derivatives for post-PCI complications, but varied in endpoint definition and cohort composition, and none compared all four scores head-to-head (34).

Comparison with existing literature underscores the incremental value of our findings. Published AUCs for early AF after STEMI cluster between 0.65 and 0.72, irrespective of whether traditional logistic regression or ensemble machine-learning approaches are used (35, 36). mC2HEST now achieves 0.786, representing an absolute gain of +0.046 to +0.169 over these benchmarks. More importantly, the IDI of 0.056 exceeds the effect sizes reported for high-sensitivity troponin or cardiac magnetic resonance parameters in similar settings, suggesting that a simple clinical score can match the prognostic resolution of biomarkers.

## 5 Limitations

First, this was a single-center retrospective study, which limits external generalizability and introduces potential selection and ascertainment bias; comorbidities were identified from discharge codes, risking misclassification of subclinical disease.

Second, AF surveillance relied on inpatient telemetry and scheduled 12-lead ECGs at 1, 3, 6, 9, and 12 months; brief asymptomatic paroxysms may have been missed, and in-hospital vs. post-discharge events and AF subtypes were not separately reported.

Third, calibration metrics (intercept, slope, Brier score) and a competing-risk analysis for death were not performed; patients who died before NOAF detection were treated as event-free, which may have slightly underestimated incidence.

Fourth, several potential confounders were not systematically collected, including left atrial diameter, LVEF, infarct size, heart failure severity grading, and serum electrolytes; residual confounding from these unmeasured factors cannot be excluded.

Fifth, treatment patterns (e.g., beta-blocker dosing, statin intensity, anti-arrhythmic use) may have evolved during the 2020–2025 study period, potentially introducing temporal heterogeneity.

Sixth, the relatively small number of NOAF events (*n* = 96) may limit the precision of subgroup estimates and the statistical power to detect modest differences between scores.

Seventh, thromboembolic events and bleeding complications were not systematically captured during follow-up; consequently, whether improved AF prediction translates into reduced stroke or net clinical benefit remains undetermined.

Eighth, missing data for key variables was 2%, and a complete-case analysis was used; although the missing rate is low, residual selection bias cannot be entirely excluded.

## 6 Conclusion

Across 1,175 consecutive STEMI patients treated by primary PCI, mC2HEST demonstrated the best discriminative performance in this cohort for predicting one-year new-onset atrial fibrillation, with an AUC of 0.786 and significant improvement in integrated discrimination compared with reference CHA_2_DS_2_-VASc-60. These findings are hypothesis-generating, and prospective multicenter validation in contemporary STEMI populations is required before routine clinical implementation.

## Data Availability

The datasets presented in this study can be found in online repositories. The names of the repository/repositories and accession number(s) can be found in the article/Supplementary Material.
